# A commentary on ‘The efficacy and safety of probiotics for prevention of chemoradiotherapy-induced diarrhea in people with abdominal and pelvic cancer: a systematic review and meta-analysis based on 23 randomized studies’

**DOI:** 10.1097/JS9.0000000000001172

**Published:** 2024-02-12

**Authors:** Qinjun Cai, Ying Shi

**Affiliations:** Department of Traditional Chinese Medicine, Beilun District People’s Hospital, Beilun Branch of the First Affiliated Hospital of Zhejiang University, Ningbo, People’s Republic of China

*Dear Editor*,

Cancers are well-known as the leading cause of death. Patients receiving radiotherapy and chemotherapy have a high risk of developing acute chemoradiotherapy-induced diarrhea^1,2^. Several strategies have been proposed to prevent and reduce chemotherapy-related diarrhea. Experimental research has indicated that the host response to cytotoxic agents is modulated by the gut microbiota. Lin and Shen^3^ performed a systematic review and meta-analysis based on 23 randomized controlled trials (RCTs), they indicated that probiotics prevented chemoradiotherapy-induced diarrhea, particularly high-grade diarrhea. Probiotics rarely cause adverse events.

In cancer patients, gut microbiota manipulation is considered to be generally safe; however, it is accompanied by risks and controversies that can potentially introduce clinical complications. The use of beneficial live microorganisms (probiotics) is among the most accepted strategies for gut microbiota modulation. A number of factors or their combinations contribute to the development of diarrhea: mucositis and consequent malabsorption, dysbiosis, immunosuppression, tight junction dysfunction, and impaired barrier function. Some cytostatics and their metabolites, such as irinotecan, induce diarrhea directly. The incidence of grade 3/4 diarrhea related to chemotherapy regimens ranges from 5 to 47%^4^. When a bolus of 5-fluorouracil (5-FU) is used in combination with irinotecan, the overall incidence of diarrhea can reach 50–80%^5^. Regarding synthetic molecules, this state is most often induced by 5-FU, capecitabine, irinotecan, and platinum derivatives. Gastrointestinal toxicity could lead to a significant impairment of the oncological patient’s quality of life.

It should be noted that there were a variety of species, strains, and doses of probiotics used, and therefore, it was difficult to come to any conclusion about the optimum probiotic strategy to use in chemoradiotherapy-induced diarrhea. Individual probiotics may differ greatly in their effects on host immune function, and the reason for their potential therapeutic efficacy remains unclear. Future studies need to establish which species, strain, and dose of probiotics are most efficacious in chemoradiotherapy-induced diarrhea. In addition, the diversity index between the studies (i.e. different types of intervention, duration, and diseases) did not allow a one-to-one comparison between the results. The included RCTs reported additional results, such as the quality of life, hospital care for bowel toxicity, neutropenia, stomatitis, and the use of anti-diarrheal drugs; however, quantitative meta-analyses were limited by the heterogeneity of their study design.

As different results for different grades of diarrhea were reported previously, further studies with Common Terminology Criteria for Adverse Events (CTCAE)^6^ grading for the outcome of diarrhea are needed. With further studies completed and available, more clinically convincing results may be drawn later. In addition, an important question of whether probiotics could lead to infection in people with cancer needs to be answered.

## Ethical approval

Not applicable.

## Consent

Not applicable.

## Sources of funding

Not applicable.

## Author contribution

Q.C.: wrote the paper; Y.S.: study design.

## Conflicts of interest disclosure

There are no conflicts of interest.

## Research registration unique identifying number (UIN)

Not applicable.

## Guarantor

Qinjun Cai.

## Data availability statement

Not applicable.

## Provenance and peer review

Not applicable.
